# Identifying Inadequate Maternal Nutrition in Pregnancies Affected by Fetal Heart Defects: A Feasibility Pilot Study Using Photo-Based Diet Quality Assessment

**DOI:** 10.3390/jcdd13030107

**Published:** 2026-02-25

**Authors:** Carson Flamm, Michelle Udine, Sarah Clauss, Anita Krishnan, Mary T. Donofrio, Michele Mietus-Snyder, Gary M. Shaw, Jennifer Klein

**Affiliations:** 1 School of Medicine and Health Sciences, The George Washington University, Washington, DC 20037, USA; 2 Division of Cardiology, Children’s National Hospital, Washington, DC 20010, USA; 3 Department of Pediatrics, Stanford University, Palo Alto, CA 94305, USA

**Keywords:** congenital heart disease, maternal nutrition, food security, diet quality

## Abstract

Etiologies of congenital heart disease (CHD) are multifactorial. The role of maternal nutrition and environmental factors among these CHD etiologies remain insufficiently understood. This pilot study evaluated the potential association between maternal diet quality, nutrient intake, and food security to fetal CHD in a cohort of 100 pregnant individuals, including 20 with CHD-affected pregnancies identified in a fetal cardiology clinic at an urban tertiary care hospital. A Diet Quality Photo Navigation (DQPN) tool assessed dietary quality and nutrient intake, while a survey collected data on demographics, health history, and food security. Comparison tests assessed for differences between CHD- and non-CHD-affected pregnancies. CHD-affected pregnancies demonstrated descriptively lower Healthy Eating Index scores, reduced prenatal multivitamin use, and lower intake of iron, manganese, fiber, and vitamin C. The non-CHD group demonstrated a significantly higher consumption of healthier snacks (*p* = 0.03), plant-based meat alternatives (*p* = 0.05), and unsweetened beverages (*p* = 0.05), while descriptively showing greater fruit and vegetable intake as compared to the CHD-affected group. No statistically significant differences in food security or socioeconomic indicators were identified. These findings demonstrate the feasibility of applying a DQPN tool in fetal health research and describe maternal dietary patterns that may inform the design of future hypothesis-driven studies. Continued investigation into maternal diet quality is critical to understand its potential role in mitigating CHD risk through targeted nutritional interventions.

## 1. Introduction

Congenital heart disease (CHD) is the most common birth defect, occurring in 1 in 100 infants born each year, and a leading cause of neonatal morbidity and mortality [1,2,3]. While genetic abnormalities are implicated in the pathogenesis of CHD, whole-genome sequencing provides genetic explanations for only 30% of CHD cases. The remaining 70% may be attributed to environmental or other potentially modifiable risk factors [4,5,6]. The maternal–fetal environment, including maternal nutrition and associated pathologies such as pregestational diabetes and hypertension, are suspected to play a prominent role in the development of the fetus.

Micronutrients are vital components of a comprehensive diet, playing crucial roles in fetal development. Maternal micronutrient irregularities are implicated in the risks of fetal CHD [7]. For example, lowered maternal folic acid intakes are associated with the risk of fetal CHD [8,9,10,11]. The vitamin A metabolite retinoic acid is crucial for embryonic heart development, and defective or excessive fetal retinoic acid signaling has been linked to cardiovascular defects [12,13]. Elevated intake of vitamin A has also been associated with increased CHD risk in some but not all studies [14]. Lower maternal levels of 25- hydroxyvitamin vitamin D have been associated with increased risks in some [15] but not all studies [16]. Across multiple studies investigating maternal nutrition and fetal heart development, diets high in one-carbon nutrients, particularly from fish, seafood and folate, have been linked with lower rates of CHD [17]. Alternatively, inadequate intake of these nutrients, especially methionine, seems to increase susceptibility to pollution-related heart defects [18]. Furthermore, after accounting for total caloric intake, maternal fat consumption showed little meaningful relationship with the development of CHD in some studies [19], highlighting the impact different nutrients may have during early cardiac development.

Numerous studies show that socioeconomically disadvantaged individuals are at higher risk for fetal CHD. Such individuals often have suboptimal nutrition and increased level of social-environmental stressors [20,21]. Our previous work demonstrated CHD clustering in disadvantaged neighborhoods [22] and showed increased usage of the Supplemental Nutrition Assistance Program (SNAP), a marker of food insecurity, to be associated with increased prevalence of fetal CHD [23]. Sociodemographic disadvantage is linked to poor diet quality [24] and inadequate nutrition [25], including insufficient micronutrients. Compared to their higher socioeconomic status counterparts, individuals with lower socioeconomic status consume fewer vegetables, fruits, whole grains, and fiber, while having a higher intake of processed meat, supporting the link between socioeconomic disparities and lower quality nutrition [26]. Similarly, those experiencing higher household food insecurity also show greater intake of ultra-processed foods, reflecting the broader financial constraints that influence dietary choices [27].

The most recent European Society for Cardiology recommendations on cardiovascular disease in pregnancy highlight maternal–fetal risk stratification as part of routine prenatal care, considering cardiometabolic and other modifiable risk factors [28]. Increasing attention is being paid to the role of maternal nutrition before, during, and after pregnancy as a modifiable determinant of cardiac risk in the maternal–fetal dyad [29]. However, despite this growing awareness, there remains need for scalable and efficient tools for dietary assessment that can be easily integrated withing existing prenatal screening.

Conventional dietary assessment tools are often time consuming, burdensome, and limited in their ability to capture real-world dietary patterns. To our knowledge, no studies have connected maternal nutritional risk and fetal CHD using Diet Quality Photo Navigation (DQPN) methods for maternal nutrition assessment. This technique allows for rapid, standardized, and scalable dietary evaluation suitable for integration into routine prenatal care. In this pilot study, we explore the feasibility of applying a DQPN approach to better clarify the multi-level influences contributing to CHD (an area where traditional nutrient-specific assessments often fall short), and to begin identifying pathways that may inform clinical and public health interventions to improve maternal–fetal risk stratification.

## 2. Materials and Methods

Participants were prospectively recruited from a fetal cardiology clinic at an urban tertiary care hospital. Women between the ages of 18 and 45 years old who presented between 18 and 34 weeks gestation were eligible. Patients with a diagnosed fetal arrhythmia and those with a discordant twin pregnancy (one fetus with CHD, one without CHD) were excluded from participation. CHD was defined as a lesion expected to need intervention, either surgical or catheter-based, within the first year of life (CHD-affected pregnancies). Those not diagnosed with such a lesion were considered as the referent group (non-affected pregnancies).

A maternal survey was used to collect basic health and demographic information, food insecurity scores and dietary quality in CHD-affected pregnancies and non-affected pregnancies. Health and demographic information collected included information such as pre-pregnancy BMI, vitamin supplementation, income and education level. Additional sociodemographic data, such as insurance type or maternal address, and health data such as fetal CHD diagnosis, were obtained from the electronic health record. Food insecurity was assessed by the validated U.S. Department of Agriculture 6-item food insecurity questionnaire creating a categorical Food Security Score (FSS). Food security status using FSS is assigned categorically as follows: scores 0–1 signify high or marginal food security; scores 2–4 signify low food security; scores 5–6 signify very low food security.

Dietary quality and nutrient intake data were derived from Diet ID [30], a validated DQPN tool designed to characterize users’ overall dietary patterns. Diet ID provides a rapid and reliable image-based assessment of diet quality across diverse populations by asking participants to choose between food images, selecting those that best-reflect their usual eating habits. Diet ID results include a Healthy Eating Index (HEI) score and average estimated intake of micro and macronutrients. These approximated nutrient outputs are derived from the patient-selected pattern of dietary patterns and do not account for supplemental nutrient intake such as from prenatal multivitamins. Supplemental vitamin use was collected separately via survey. The HEI is a standardized and validated measure of diet quality that assesses the alignment of a given dietary intake with the key recommendations and dietary patterns published in the *Dietary Guidelines for Americans* [31,32]. HEI is scaled from 0 to 100, with higher scores indicating higher dietary quality. Diet ID also generated a second metric, the Diet ID score, reported as an integer scale from 1 to 10. This score is derived from HEI analysis and adjusted for variation across dietary patterns, providing an alternative estimate of overall diet quality. Additionally, Diet ID correlates well with traditional dietary assessment methods, including 24 h dietary recall and food frequency questionnaires [33]. Diet ID is shown to be comparable to these established methods, but also offers potential advantages such as improved efficiency, reduced respondent burden, and real-time analysis [34]. The survey was available in English or Spanish.

We conducted statistical analysis using R to investigate the relationship between maternal nutrition and CHD. Our analysis utilized a combined dataset of survey, electronic health record, and diet quality data, which was processed and cleaned in R. We generated summary statistics to compare the characteristics of maternal–fetal dyads with and without CHD. We then fit both univariable and multivariable logistic regression models to estimate the associations between CHD and various predictors, including diet quality scores, food security scores, prenatal multivitamin use, and other maternal characteristics.

Because this feasibility pilot study included a relatively small sample size of 100 participants, analyses were performed to outline dietary patterns and inform future hypothesis-driven research and did not aim to prove definitive statistical associations. Statistical significance was defined as *p*-values < 0.05. Highlighted results that did not meet this cutoff are shown descriptively to present observed differences between comparison groups, and to identify areas for investigation in subsequent adequately powered studies. The primary purpose of these findings is to show how the Diet ID/DQPN method can be utilized to identify aspects of diet and nutrition warranting follow-up with larger studies in the future.

## 3. Results

The analyses shared in this section, including quantitative nutrient and food group studies, are presented to describe dietary patterns and notable differences between CHD and non-CHD groups in this pilot cohort and not to establish causal relationships. Of the 100 participants enrolled in the study, 20 (20.0%) were classified as CHD-affected pregnancies, while 80 (80.0%) had non-CHD pregnancies. Maternal demographics and clinical characteristics (Table 1) were largely similar between groups. Gestational age at fetal echocardiography was higher in the CHD group (28.1 vs 24.8 weeks, *p* < 0.01). Primary indication for fetal echocardiography differed significantly (*p* < 0.01), with referrals for CHD pregnancies most often due to fetal anomaly or genetic abnormality, while non-CHD referrals were more frequently for maternal history of in vitro fertilization. Overall, 23.0% of participants had a diagnosis of pre-gestational diabetes, with 25.0% of the non-CHD group and 15.0% of the CHD group affected (*p* = 0.55). Due to the small number of CHD pregnancies with pre-gestational diabetes (*n* = 3), there was an insufficient sample to assess differences in diabetes prevalence between the CHD and non-CHD pregnancies.

Maternal dietary quality, as measured by the HEI and Diet ID scores, was lower among CHD pregnancies compared to non-CHD pregnancies, though differences did not reach statistical significance. The average HEI score for CHD pregnancies was 55.67, compared to 68.47 for non-CHD pregnancies (*p* = 0.08), while the average Diet ID score for CHD pregnancies was 5.33, compared to 6.56 for non-CHD pregnancies (*p* = 0.13). Prenatal multivitamin use was also descriptively lower in the CHD group (85.0%) compared to the non-CHD group (96.3%, *p* = 0.09). There was no significant difference in food security, measured using Food Security Scores (FSS), between CHD pregnancies compared to non-CHD pregnancies. Average maternal BMI was descriptively higher in CHD pregnancies compared to non-CHD pregnancies, though this difference was not statistically significant.

We comprehensively evaluated maternal nutrient intake as assessed by Diet ID (Appendix A Table A1). Selected nutrients considered clinically or biologically relevant are highlighted in Table 2. Though dietary quality assessment using Diet ID did not reveal any statistically significant differences in nutrient intake between the mothers of CHD and non-CHD pregnancies, descriptive differences were observed across several macronutrient and micronutrient domains (Table 2).

Diet ID analysis of estimated daily food group intake revealed several observed differences between women with a CHD vs. non-CHD pregnancy, describing dietary patterns that may inform hypotheses for future studies. The complete analysis is found in Appendix A Table A2 and selected food groups considered clinically relevant are highlighted in Table 3. Non-CHD pregnancies showed significantly higher daily consumption of healthier snacks, plant-based meat alternatives, and unsweetened beverages as compared to CHD-affected pregnancies (*p* = 0.03, *p* = 0.05, and *p* = 0.05 respectively). Additional descriptive differences were observed across several dietary subgroups (Table 3).

Univariable logistic regression (Table 4) showed that higher HEI scores and prenatal multivitamin use were observed in relation to lower odds of CHD-affected pregnancy, while maternal education ending at high school was noted alongside higher odds of CHD-affected pregnancy, though these findings did not meet the predefined threshold for statistical significance. A significant association between later gestational age and CHD was present in the univariable analysis that persisted in a multivariable analysis adjusting for dietary, socioeconomic, and demographic features. Given that no further independent associations emerged in this multivariable analysis, likely due to limited study power, the full multivariable results are reported in Appendix B Table A3.

**Figure 1:**
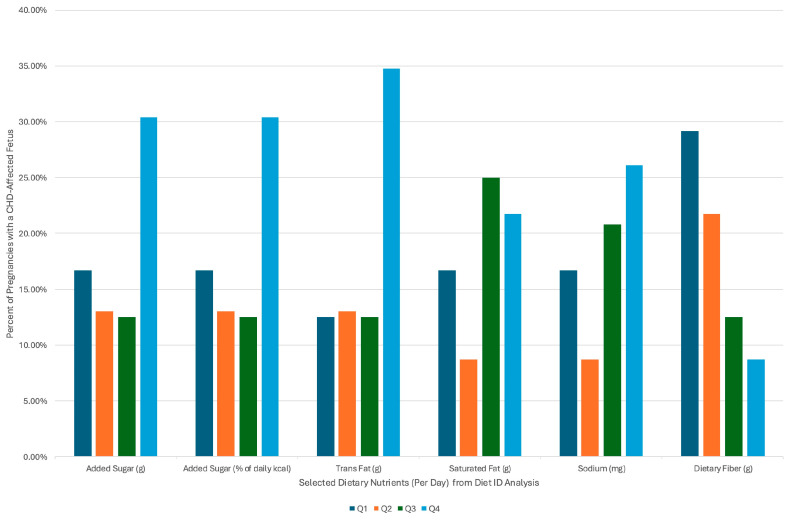
Proportion of CHD-affected pregnancies in each quartile of selected ultra-processed food-associated nutrients.

The intakes of key macronutrients, particularly those associated with ultra-processed foods (UPF), were divided into quartiles, and the percentage of those in each quartile to have CHD are displayed in Figure 1. There was a higher proportion of CHD in the top quartile (Q4) for added sugars (*p* = 0.39), added sugars as a percent of daily calories (*p* = 0.39), trans fats (*p* = 0.19), and sodium (*p* = 0.50) compared to lower quartiles. For saturated fat, the proportion of CHD pregnancies was higher in Q3 and Q4 compared to Q1 and Q2 (*p* = 0.52). Dietary fiber showed the opposite pattern, with a greater percentage of Q1 having CHD as compared to Q4 (*p* = 0.27). These differences are descriptive and did not reach statistical significance by Fischer’s Exact Test, but could help guide future investigation.

## 4. Discussion

First and foremost, this pilot study establishes the feasibility of assessing maternal dietary habits and food security through validated survey and use of a novel Dietary Photo Navigation Tool. Participants recruited to the study were approached consecutively in the Children’s National fetal cardiology clinic. The significantly higher gestational age at time of echocardiogram observed in the CHD cohort is most likely reflective of differences in recruitment patterns rather than clinical or biological distinctions between the groups. Non-CHD participants typically were recruited around 20–24 weeks’ gestation, at the time of initial fetal echocardiogram. For those pregnancies found to be affected by fetal CHD, most women elected study participation at a first follow-up visit (typically 1 month after diagnosis) rather than at the time of receiving the diagnosis, and thus a higher average gestational age was seen in this cohort. Surprisingly, no differences were observed in several key socioeconomic markers—including education level, insurance type, income, and food security scores—between CHD and non-CHD pregnancies. This contrasts with prior research that has shown higher CHD birth prevalence in low-income and medically underserved regions, where inadequate resources, limited insurance coverage, and insufficient screening have been implicated as contributors to fetal CHD risk [35,36]. This may again reflect limited statistical power with a small sample size but could also be influenced by referral patterns. Because recruitment occurred at Children’s National Hospital, a large tertiary care center primarily serving patients referred for specialized care, both CHD and non-CHD participants may represent families with sufficient resources to access specialized healthcare, possibly reducing hypothesized socioeconomic differences between the groups.

This study, even with its small size, observed descriptive associations between suboptimal maternal nutrition and fetal CHD, including for micro- and macronutrient intake and composite dietary quality scores. Though these differences fell short of the predetermined threshold for statistical significance, they suggest potential trends valuable for hypothesis generation for future studies. Specifically, higher intake of added sugars or trans fats and lower fiber intake are associated with ultra-processed foods, thus raising the question of whether maternal consumption of these dietary factors is a risk factor for fetal CHD. To our knowledge, no studies have explored possible associations between maternal consumption of processed foods and the development of fetal CHD, highlighting a potential area for future research [37,38].

Additionally, the lack of significant differences in vitamin A, vitamin D, folate, and other micronutrients such as iron and B vitamins contrasts with the existing literature and may be a result of the imprecision and insufficient statistical power of this pilot study. Food security was also not associated with fetal CHD. Limited statistical power may also explain this lack of association.

Our data show a descriptive disparity in reported prenatal multivitamin use between CHD and non-CHD affected pregnancies that did not meet the threshold for significance. Although these findings are preliminary and not intended to establish causation, they suggest potential for future investigations into relationships between prenatal vitamin use and the development of CHD. Prenatal folic acid supplementation has been strongly linked with a decreased incidence of several congenital heart defects, while evidence for the protective effects of prenatal multivitamin use remains mixed [39,40,41]. Within this context, our results aim first and foremost to inform the design of future studies exploring overall maternal dietary quality as it relates to CHD risk.

This pilot study has a few notable limitations. Most prominent is its small size. Additionally, we relied on self-reported survey data, which could potentially introduce biases based on recall bias or social desirability responses. Such errors in reporting may have affected reported dietary intake, socioeconomic status, and health-related behaviors. Furthermore, the study population reflects a consecutively recruited referral-based cohort who met clinical indications to receive a fetal echocardiogram at a tertiary care center. As such, there may be systematic differences in demographics, healthcare access, and clinical complexity distinguishing this group from the broader maternal population and limiting the external validity of our findings, emphasizing the need for future large-cohort studies in more varied clinical settings. Additionally, this feasibility study includes exploratory comparative data unadjusted for multiple comparisons, increasing the risk of type 1 error. As such, all non-significant findings are presented descriptively with the intention of guiding hypothesis generation for future investigation. Addressing these limitations in future research through large, longitudinal studies with comprehensive dietary assessments and direct measurements of nutritional status will be essential to further elucidate the relationship between maternal nutrition and fetal CHD risk.

## 5. Conclusions

We demonstrate the feasibility of using a novel approach to collecting maternal nutrition data in pregnancies with and without CHD. Although our sample size is modest, these findings reinforce the need to further study maternal nutrition as a potentially important contributor to the development of CHD. Equally important, this pilot study shows that DQPN methods like Diet ID function well in a clinical setting and that DietID is quick for patients, requires minimal staff time, and can be readily scaled as enrollment grows. These findings showcase the feasibility of utilizing DQPN technology in a fetal cardiology clinic and outline preliminary patterns in maternal diet that could help guide the development of further adequately powered, hypothesis-driven research. This work serves as a foundation for larger prospective investigations aimed at understanding the complex relationships between maternal nutrition and fetal cardiovascular development.

## Figures and Tables

**Table 1:** Study participant demographics.

Variable	Non-CHD (Mean [SD])	CHD (Mean [SD])	*p*-Value
Age (yr) (*n* = 100)	33.53 [5.50]	32.35 [5.38]	0.39
Gestational Age (weeks) (*n* = 100)	24.78 [3.33]	28.13 [3.73]	<0.01
Height (in) (*n* = 95)	64.51 [2.78]	63.42 [1.92]	0.05
Weight (lb) (*n* = 96)	176.81 [54.29]	186.74 [54.12]	0.48
Body Mass Index (lb/in^2) (*n* = 95)	29.83 [8.72]	32.62 [9.30]	0.25
Diet ID Average Score (*n* = 95)	6.56 [2.71]	5.33 [2.94]	0.13
HEI Average Score (*n* = 95)	68.47 [24.62]	55.67 [26.60]	0.08
**Variable**	**Non-CHD**	**CHD**	***p*-Value**
Ethnicity			
Hispanic or Latino	25.00% [20/80]	27.78% [5/18]	0.77
Not Hispanic or Latino	75.00% [60/80]	72.22 [13/18]	
Race			
American Indian or Alaskan Native	0.00% [0/80]	5.00% [1/20]	0.82
Asian	3.75% [3/80]	5.00% [1/20]	
Black or African American	32.50% [26/80]	40.00% [8/20]	
Middle Eastern or North African	1.25% [1/80]	0.00% [0/20]	
Native Hawaiian or Other Pacific Islander	0.00% [0/80]	0.00% [0/20]	
White	42.50% [34/80]	40.00% [8/20]	
Other	20.00% [16/80]	10.00% [2/20]	
Employment			
Full-Time	64.56% [51/79]	60.00% [12/20]	0.92
Part-Time	6.33% [5/79]	5.00% [1/20]	
Seeking	5.06% [4/79]	10.00% [2/20]	
Stay-At-Home Parent	18.99% [15/79]	25.00% [5/20]	
Student	2.53% [2/79]	0.00% [0/20]	
Other (unemployed)	2.53% [2/79]	0.00% [0/20]	
Highest Level of Education			
Less than high school	7.5% [6/80]	5.00% [1/20]	0.57
High school	30.00% [24/80]	50.00% [10/20]	
Technical or trade school	1.25% [1/80]	0.00% [0/20]	
Two-year degree from a college or university	6.25% [5/80]	5.00% [1/20]	
Four-year degree from a college of university	25.00% [20/80]	10.00% [2/20]	
Postgraduate degree	30.00% [24/80]	30.00% [6/20]	
Marital Status			
Single (never married)	13.75% [11/80]	15.00% [3/20]	0.88
Living with partner	21.25% [17/80]	20.00% [4/20]	
Married	62.50% [50/80]	60.00% [12/20]	
Divorced/Separated	2.50% [2/80]	5.00% [1/20]	
Total Household Income			
Less than $15,000	12.66% [10/79]	15.79% [3/19]	0.29
$15,000-$29,999	12.66% [10/79]	10.53% [2/19]	
$30,000-$44,999	6.33% [5/79]	15.79% [3/19]	
$45,000-$59,999	6.33% [5/79]	10.53% [2/19]	
$60,000-$79,999	7.59% [6/79]	10.53% [2/19]	
$80,000-$99,999	2.53% [2/79]	10.53% [2/19]	
$100,000-$149,999	7.59% [6/79]	5.26% [1/19]	
$150,000 or above	44.30% [35/79]	21.06% [4/19]	
Insurance Type			
Public	33.75% [27/80]	45.00% [9/20]	0.55
Private	65.00% [52/80]	55.00% [11/20]	
Unknown	1.25% [1/80]	0.00% [0/20]	
Pregestational Diabetes			
Yes	25.00% [20/80]	15.00% [3/20]	0.55
No	75.00% [60/80]	85.00% [17/20]	
Activity Level			
Minimal	18.42% [14/76]	17.65% [3/17]	0.98
Light	39.49% [30/76]	35.29% [6/17]	
Moderate	28.95% [22/76]	29.41% [5/17]	
Active	13.16% [10/76]	17.65% [3/17]	
Food Security Score			
0–1 (High or marginal food security)	75.00% [60/80]	85.00% [17/20]	0.82
2–4 (Low food security)	16.25% [13/80]	10.00% [2/20]	
5 (Very low food security)	8.75% [7/80]	5.00% [1/20]	
Prenatal Multivitamin Use			
Yes	96.25% [77/80]	85.00% [17/20]	0.09
No	3.75% [3/80]	15.00% [3/20]	
Primary Indication for Fetal Echo			
Insufficient Screening US	10.00% [8/80]	5.00% [1/20]	<0.01
Family History	3.75% [3/80]	0.00% [0/20]	
Fetal Anomaly	17.50% [14/80]	45.00% [9/20]	
Genetic Abnormality	8.75% [7/80]	35.00% [7/20]	
IVF	25.00% [20/80]	10.00% [2/20]	
Maternal Health History	35.00% [28/80]	5.00% [1/20]	

**Table 2:** Average intake of selected nutrients in CHD vs. non-CHD pregnancies.

Nutrient Type	Average Intake (SD) Non-CHD (*n* = 77)	Average Intake (SD) CHD (*n* = 18)	*p*-Value
Macronutrients			
Dietary Fiber (g)	35.92 (20.46)	27.48 (19.04)	0.11
Trans Fats (g)	1.54 (1.40)	2.17 (1.57)	0.14
Added Sugars (g)	44.36 (51.82)	64.40 (61.93)	0.22
Estimated Calorie Intake (kcal)	2432.74 (452.51)	2428.30 (333.36)	0.96
Micronutrients			
Iron (mg)	19.21 (5.29)	17.02 (5.05)	0.11
Lycopene (µg)	7559.05 (4016.68)	6362.74 (2787.16)	0.14
Manganese (mg)	6.30 (3.30)	4.79 (3.31)	0.09
Methionine (g)	2.35 (1.05)	2.36 (1.06)	0.98
Sodium (mg)	3269.83 (1395.07)	3663.85 (1382.38)	0.29
Vitamins			
Vitamin A (RAE µg)	1535.86 (920.74)	1257.98 (878.47)	0.24
Total Folate (B9) (mcg)	595.26 (245.92)	521.01 (246.23)	0.26
Thiamin (B1) (mg)	2.06 (0.49)	2.06 (0.75)	0.99
Riboflavin (B2) (mg)	2.46 (0.65)	2.42 (0.73)	0.82
Niacin (B3) (mg)	30.04 (11.02)	28.87 (10.16)	0.67
Pantothenic Acid (B5) (mg)	6.95 (2.10)	6.42 (2.16)	0.36
Vitamin B6 (mg)	2.73 (1.08)	2.48 (1.10)	0.39
Vitamin B12 (mcg)	5.32 (3.17)	5.44 (3.33)	0.89
Vitamin C (mg)	179.60 (120.19)	133.38 (114.03)	0.14
Vitamin D (mcg)	6.28 (4.29)	6.36 (4.54)	0.95

**Table 3:** Daily serving size equivalents of selected food groups in CHD vs. non-CHD pregnancies.

Food Group	Standard Daily Serving Size, Examples	Standard Daily Serving Size Equivalents Non-CHD (*n* = 77)	Standard Daily Serving Size Equivalents CHD (*n* = 18)	*p*-Value
Fried Foods	1 oz fried fish, chicken, chicken-fried steak; 1 oz fried snack chips; 1 oz pork rinds	0.63 (0.90)	1.00 (1.12)	0.21
Fruit	1/2 cup equivalent	2.59 (2.14)	1.76 (2.13)	0.15
Full-Fat Dairy Products	1 cup milk; 1 1/2 oz natural cheese; 2 oz processed cheese; 2 cups cottage cheese; 1 cup yogurt	0.44 (0.52)	0.67 (0.97)	0.34
Healthier Snacks	1 oz whole grain snack bar; 1 oz whole-grain snack chips; 1 oz plain popped popcorn	0.59 (1.00)	0.26 (0.39)	0.03
Plant-Based Meat Alternatives	1 oz tofu or tempeh; 1 oz meat substitute; 1/2 oz soy nuts	0.71 (0.96)	0.37 (0.55)	0.05
Sweetened Beverages	8 fl oz coffee, tea; 1 fl oz soft drinks; 8 fl oz sweet tea, lemonade, fruit drinks; 8 fl oz sweet sports drink	0.68 (1.26)	1.43 (1.72)	0.09
Unsweetened Beverages	8 fl oz plain coffee, tea; 8 fl oz seltzer water or club soda; 8 fl oz water	2.01 (1.09)	1.44 (1.05)	0.05
Vegetables	1/2 cup chopped raw; 1/2 cup cooked; 1 cup raw leafy; 1 cup raw large chunks; 1 medium potato	7.98 (5.38)	5.90 (5.00)	0.13
Whole Grains	1 medium slice of bread (1 oz); 6 inch tortilla; 1 oz tortilla chips; 1 small roll; 1/2 cup cooked grains or pasta; 1 oz dry cereal; 1/2 cup hot cereal; 1 oz crackers	2.41 (2.48)	1.45 (2.42)	0.15

Values reported as average serving size equivalents (standard deviation).

**Table 4:** Maternal–fetal dyad associations with fetal CHD: univariable analyses.

Characteristic	*n*	Univariable OR (95% CI)	*p*-Value
Diet Quality			
Diet ID Score	94	0.86 (0.71, 1.03)	0.11
HEI	94	0.98 (0.96, 1.00)	0.07
Food Security	100		
Food Secure		Reference	
Low Food Security		0.54 (0.08, 2.23)	0.40
Very Low Food Security		0.50 (0.03, 3.12)	0.50
Prenatal Vitamin	100		
No		Reference	
Yes		0.22 (0.04, 1.28)	0.08
Diabetes	100		
No		Reference	
Yes		0.53 (0.12, 1.79)	0.30
Body Mass Index	94	1.03 (0.98, 1.09)	0.20
Gestational Age	100	1.28 (1.12, 1.48)	<0.01
Race	100		
Black		Reference	
Multiracial/Other		0.60 (0.14, 2.17)	0.40
White		0.76 (0.25, 2.33)	0.60
Ethnicity	98		
Hispanic/Latina		Reference	
Not Hispanic/Latina		0.87 (0.29, 2.97)	0.80
Marital Status	100		
Div/Sep		Reference	
Living with Partner		0.47 (0.04, 11.6)	0.60
Married		0.48 (0.04, 10.8)	0.60
Single		0.55 (0.04, 14.1)	0.70
Education	100		
Four Year		Reference	
High School		4.17 (0.96, 29.2)	0.09
Less than HS		1.67 (0.07, 20.6)	0.70
Postgrad		2.50 (0.51, 18.4)	0.30
Technical/Trade		—	>0.99
Two Year		2.00 (0.08, 25.5)	0.60
Employment	99		
Full-time		Reference	
Other (Unemployed)		—	>0.99
Part-time		0.85 (0.04, 5.95)	0.90
Seeking Employment		2.13 (0.27, 12.3)	0.40
Stay at Home Parent		1.42 (0.40, 4.52)	0.60
Student		—	>0.99
Income ($k)	98		
<15		Reference	
15–29		0.67 (0.08, 4.88)	0.70
30–44		2.00 (0.28, 14.8)	0.50
45–59		1.33 (0.14, 10.9)	0.80
60–79		1.11 (0.12, 8.75)	>0.99
80–99		3.33 (0.30, 40.3)	0.30
100–149		0.56 (0.02, 5.57)	0.60
>150		0.38 (0.07, 2.19)	0.30
Age (years)	100	0.96 (0.88, 1.05)	0.40

## Data Availability

The data presented in this study are available upon request from the corresponding author due to privacy reasons.

## Abbreviations

The following abbreviations are used in this manuscript:

CHD	Congenital Heart Disease
DQPN	Diet Quality Photo Navigation
FSS	Food Security Score
HEI	Healthy Eating Index
RAE	Retinol Activity Units
SD	Standard Deviation
SNAP	Supplemental Nutrition Assistance Program
UPF	Ultra-Processed Food

## Appendix A

### Appendix A.1

**Table A1:** Average nutrient intake of CHD vs. non-CHD pregnancies.

Nutrient	Average Intake (SD) Overall (*n* = 95)	Average Intake (SD) Non-CHD (*n* = 77)	Average Intake (SD) CHD (*n* = 18)	*p*-Value
Added Sugars (g)	48.16 (54.10)	44.36 (51.82)	64.40 (61.93)	0.22
Alanine (g)	5.19 (2.28)	5.21 (2.27)	5.09 (2.38)	0.85
Alpha-Carotene (µg)	1529.74 (2054.69)	1598.07 (2120.80)	1237.44 (1767.61)	0.46
Arginine (g)	6.16 (2.62)	6.20 (2.60)	5.95 (2.81)	0.73
Aspartic Acid (g)	9.94 (4.00)	10.04 (3.97)	9.51 (4.23)	0.64
Beta-Carotene (µg)	11,815.88 (9895.48)	12,419.00 (9876.90)	9235.84 (9829.91)	0.23
Calcium (mg)	1112.84 (333.17)	1131.40 (328.64)	1033.44 (350.29)	0.29
Cholesterol (mg)	340.29 (221.13)	328.79 (213.90)	389.50 (250.34)	0.35
Choline (mg)	465.95 (186.39)	466.72 (184.18)	462.68 (201.07)	0.94
Copper (mg)	1.98 (0.96)	2.03 (0.96)	1.77 (0.95)	0.30
Cystine (g)	1.43 (0.49)	1.44 (0.49)	1.39 (0.50)	0.69
Glutamic Acid (g)	20.06 (5.91)	20.18 (5.87)	19.54 (6.24)	0.70
Glycine (g)	4.52 (1.96)	4.53 (1.94)	4.45 (2.08)	0.87
Histidine (g)	2.86 (1.22)	2.87 (1.21)	2.85 (1.28)	0.96
Iron (mg)	18.79 (5.29)	19.21 (5.29)	17.02 (5.05)	0.11
Isoleucine (g)	4.66 (1.87)	4.67 (1.85)	4.61 (1.98)	0.90
Leucine (g)	8.12 (3.11)	8.15 (3.10)	7.98 (3.23)	0.85
Lutein + Zeaxanthin (µg)	8541.45 (10,398.45)	8804.94 (10,035.88)	7414.33 (12,081.01)	0.66
Lycopene (µg)	7332.38 (3830.32)	7559.05 (4016.68)	6362.74 (2787.16)	0.14
Lysine (g)	7.12 (3.41)	7.12 (3.42)	7.10 (3.50)	0.98
Magnesium (mg)	463.94 (233.14)	480.64 (232.17)	392.49 (230.01)	0.16
Manganese (mg)	6.01 (3.34)	6.30 (3.30)	4.79 (3.31)	0.09
Methionine (g)	2.35 (1.05)	2.35 (1.05)	2.36 (1.06)	0.98
Net/Available Carbs (g)	242.56 (85.27)	242.79 (85.15)	241.56 (88.21)	0.96
Niacin (B3) (mg)	29.82 (10.82)	30.04 (11.02)	28.87 (10.16)	0.67
Omega-3 Fat (g)	3.58 (2.18)	3.64 (2.17)	3.34 (2.29)	0.62
Pantothenic Acid (mg)	6.85 (2.11)	6.95 (2.10)	6.42 (2.16)	0.36
Phenylalanine (g)	4.63 (1.59)	4.66 (1.58)	4.51 (1.68)	0.74
Phosphorus (mg)	1684.86 (481.09)	1712.79 (479.69)	1565.41 (482.08)	0.25
Potassium (mg)	3996.15 (1633.24)	4096.19 (1626.39)	3568.22 (1638.62)	0.23
Proline (g)	6.27 (1.68)	6.29 (1.67)	6.19 (1.80)	0.84
PUFA 18:3 n-3 (alpha-linolenic acid [ALA]) (g)	3.10 (1.81)	3.16 (1.82)	2.86 (1.80)	0.54
PUFA 20:5 (eicosapentaenoic acid [EPA]) (g)	0.10 (0.13)	0.10 (0.13)	0.10 (0.14)	0.90
PUFA 22:6 (docosahexaenoic acid [DHA]) (g)	0.30 (0.40)	0.30 (0.40)	0.29 (0.39)	0.96
Riboflavin (B2) (mg)	2.46 (0.66)	2.46 (0.65)	2.42 (0.73)	0.82
Selenium (µg)	153.47 (50.05)	154.81 (50.98)	147.72 (46.77)	0.57
Serine (g)	4.86 (1.67)	4.89 (1.65)	4.73 (1.76)	0.72
Sodium (mg)	3344.48 (1394.00)	3269.83 (1395.07)	3663.85 (1382.38)	0.29
Thiamin (B1) (mg)	2.06 (0.55)	2.06 (0.49)	2.06 (0.75)	0.99
Threonine (g)	4.17 (1.72)	4.19 (1.71)	4.08 (1.80)	0.82
Carbohydrates (g)	277.22 (86.79)	279.01 (87.62)	269.56 (85.14)	0.68
Dietary Fiber (g)	34.32 (20.38)	35.92 (20.46)	27.48 (19.04)	0.11
Total Fat (g)	104.40 (30.86)	103.90 (31.55)	106.53 (28.47)	0.73
Total Folate (µg)	581.19 (246.41)	595.26 (245.92)	521.01 (246.23)	0.26
Monounsaturated Fat (g)	43.10 (18.54)	43.47 (19.19)	41.49 (15.82)	0.65
Polyunsaturated Fat (g)	25.33 (7.29)	25.61 (7.50)	24.14 (6.37)	0.40
Protein (g)	107.91 (39.44)	108.54 (39.23)	105.23 (41.36)	0.76
Saturated Fat (g)	27.42 (13.60)	26.34 (12.62)	32.03 (16.85)	0.19
Total Sugars (g)	105.55 (39.29)	104.16 (38.74)	111.48 (42.22)	0.51
Trans Fat (g)	1.66 (1.45)	1.54 (1.40)	2.17 (1.57)	0.14
Vitamin A (RAE µg)	1483.21 (914.87)	1535.86 (920.74)	1257.98 (878.47)	0.24
Tryptophan (g)	1.25 (0.45)	1.26 (0.45)	1.20 (0.45)	0.61
Tyrosine (g)	3.52 (1.35)	3.52 (1.35)	3.50 (1.40)	0.95
Valine (g)	5.26 (1.98)	5.28 (1.97)	5.17 (2.09)	0.84
Vitamin B12 (µg)	5.34 (3.18)	5.32 (3.17)	5.44 (3.33)	0.89
Vitamin B6 (mg)	2.68 (1.08)	2.73 (1.08)	2.48 (1.10)	0.39
Vitamin C (mg)	170.84 (119.84)	179.60 (120.19)	133.38 (114.03)	0.14
Vitamin D (µg)	6.30 (4.32)	6.28 (4.29)	6.36 (4.54)	0.95
Vitamin E (mg)	17.10 (8.88)	17.35 (8.83)	16.01 (9.25)	0.58
Vitamin K (µg)	463.88 (521.57)	479.62 (506.26)	396.55 (593.67)	0.59
Zinc (mg)	13.31 (3.76)	13.38 (3.60)	13.02 (4.50)	0.76
Estimated Calorie Intake (kcal)	2431.90 (430.88)	2432.74 (452.51)	2428.30 (333.36)	0.96
Added Sugar % of Daily Calories (%)	7.89 (8.37)	7.26 (7.88)	10.57 (10.03)	0.20
Percent Carbohydrates (%)	45.70 (11.80)	45.97 (11.55)	44.58 (13.11)	0.68
Percent Total Fat (%)	38.55 (8.32)	38.35 (8.19)	39.43 (9.04)	0.65
Percent Protein (%)	17.72 (5.36)	17.83 (5.18)	17.27 (6.23)	0.73

### Appendix A.2

**Table A2:** Daily serving size equivalents of food groups in CHD vs. non-CHD pregnancies.

Food Group	Standard Daily Serving Size	Standard Daily Serving Size Equivalents Entire Population (*n* = 95)	Standard Daily Serving Size Equivalents, Non-CHD (*n* = 77)	Standard Daily Serving Size Equivalents, CHD (*n* = 18)	*p*-Value
Alcoholic Beverages	12 fl oz beer 5 fl oz wine 1 1/2 fl oz liquor	0.15 (0.21)	0.13 (0.20)	0.20 (0.23)	0.28
Artificially Sweetened Beverages	8 fl oz (1 cup) coffee, tea with artificial sweetener 8 fl oz (1 cup) diet soft drinks 8 fl oz (1 cup) diet tea, lemonade, fruit drinks, sports drinks	0.60 (0.89)	0.57 (0.85)	0.74 (1.08)	0.52
Beans Lentils	1/2 cup cooked (canned or from dried) 1/3 cup hummus or bean dip	0.51 (0.70)	0.54 (0.71)	0.39 (0.64)	0.37
Dairy Based Desserts	1 cup pudding 1/2 cup ice cream or frozen yogurt	0.08 (0.19)	0.08 (0.20)	0.07 (0.17)	0.80
Eggs	1 large egg 2 egg whites	0.80 (0.60)	0.78 (0.57)	0.87 (0.72)	0.61
Fatty Condiments	1 tsp oil 1 tbsp cream 1 tbsp salad dressing 1 tbps mayonnaise	0.61 (0.72)	0.60 (0.72)	0.64 (0.74)	0.83
Fish	1 oz fish or shellfish	1.40 (1.88)	1.46 (1.95)	1.16 (1.58)	0.50
Fried Foods	1 oz fried fish, chicken, or chicken fried steak 1 oz fried snack chips 1/2 cup chopped French Fries or other fried veggie	0.70 (0.95)	0.63 (0.90)	1.00 (1.12)	0.21
Fried Vegetables	1/2 cup chopped French Fries or other fried veggie	0.43 (0.65)	0.41 (0.66)	0.49 (0.59)	0.64
Fruit	1/2 cup sliced or chopped 1 medium handheld fruit 1/4 cup dried fruit	2.43 (2.15)	2.59 (2.14)	1.76 (2.13)	0.15
Fruit Juice	1/2 cup 100% fruit juice	0.21 (0.30)	0.23 (0.32)	0.13 (0.22)	0.10
Full Fat Dairy Products	1 cup milk 1 1/2 oz natural cheese 2 oz processed cheese 2 cups cottage cheese 1 cup yogurt	0.48 (0.63)	0.44 (0.52)	0.67 (0.97)	0.34
Healthier Snacks	1 oz whole-grain snack bar 1 oz whole-grain snack chips 1 oz plain popped popcorn	0.53 (0.93)	0.59 (1.00)	0.26 (0.39)	0.03
Nuts Seeds	1/2 oz nuts or seeds 1 tbsp nut or seed butter	1.64 (1.62)	1.70 (1.61)	1.37 (1.68)	0.46
Plant-Based Dairy Alternatives	1 oz tofu or tempeh 1 oz meat substitute 1/2 oz soy nuts	0.54 (0.83)	0.47 (0.72)	0.80 (1.16)	0.26
Plant-Based Meat Alternatives	1 oz tofu or tempeh 1 oz meat substitute 1/2 oz soy nuts	0.64 (0.90)	0.71 (0.96)	0.37 (0.55)	0.05
Poultry	1 oz “poultry lean poultry fried chicken” 1/2 oz jerky	1.93 (2.43)	1.94 (2.50)	1.85 (2.19)	0.88
Processed Fruits	1/2 cup fried fruits; fruit based savory snack	0.02 (0.08)	0.02 (0.07)	0.03 (0.09)	0.52
Processed Meat	1 oz “cold cuts and sausage lean cold cuts and sausage cured pork lean cured pork meat based savory snack”	0.84 (1.12)	0.77 (1.08)	1.13 (1.26)	0.27
Red Meat	“1 oz “beef lean beef veal lean veal lamb lean lamb fresh pork lean fresh pork game organ meats” 1/2 oz jerky”	1.66 (2.26)	1.54 (2.17)	2.16 (2.64)	0.37
Reduced Or Non-Fat Dairy Products	“1 cup milk 1 1/2 oz natural cheese 2 oz processed cheese 2 cups cottage cheese 1 cup yogurt”	1.07 (0.68)	1.09 (0.68)	0.99 (0.70)	0.59
Refined Grains	“1 medium slice of bread (1 oz) 6 inch tortilla 1 oz tortilla chips 1 small roll 1/2 cup cooked grains or pasta 1 oz dry cereal 1/2 cup hot cereal 1 oz crackers”	4.52 (3.71)	4.45 (3.63)	4.83 (4.15)	0.72
Salty Snacks	1 oz snack chips 1 oz buttered or flavored popcorn 1 oz pork rinds	0.30 (0.50)	0.26 (0.45)	0.46 (0.66)	0.24
Saturated Unhealthy Fats	1 tsp butter, hard margarine, or shortening 1 tsp lard or tallow 1 tbsp creamy dressings and condiments	1.97 (2.18)	1.81 (1.95)	2.62 (2.96)	0.28
Shellfish	1 oz “shellfish fried shellfish” (without shell)	0.25 (0.56)	0.22 (0.54)	0.35 (0.66)	0.46
Sweet Salty Condiments	“1oz sweet/salty sauces syrup, honey, jam sugar fudge, caramel, butterscotch, etc. frosting or icing”	3.11 (3.18)	3.00 (3.21)	3.56 (3.08)	0.50
Sweetened Beverages	“8 fl oz (1 cup) coffee, tea 8 fl oz (1 cup) soft drinks 8 fl oz (1 cup) sweet tea, lemonade, fruit drinks 8 fl oz (1 cup) sweet sports drinks”	0.82 (1.38)	0.68 (1.26)	1.43 (1.72)	0.09
Sweets Desserts	1/2 cup frozen dessert 1 cup pudding 1 average piece of pie or cake 1 medium donut, Danish, cookie, brownie, or pastry 1 average snack bar or quickbread 1 tsp sugar 2 tbsp syrup 1 1/2 oz candy or chocolate 2 tbsp fudge or caramel 2 tbsp frosting or icing	3.30 (3.30)	3.17 (3.33)	3.85 (3.23)	0.43
Unsaturated Healthier Fats	1 tsp vegetable oil 1/2 cup chopped avocado	5.71 (5.00)	5.84 (5.11)	5.18 (4.58)	0.59
Unsweetened Beverages	8 fl oz (1 cup) plain coffee, tea 8 fl oz (1 cup) seltzer water or club soda 8 fl oz (1 cup) water	1.90 (1.10)	2.01 (1.09)	1.44 (1.05)	0.05
Vegetable Juice	1/2 cup vegetable juice	0.00 (0.00)	0.00 (0.00)	0.00 (0.00)	N/A
Vegetables	1/2 cup chopped raw 1/2 cup cooked 1 cup raw leafy 1 cup raw large chunks 1 medium potato	7.59 (5.35)	7.98 (5.38)	5.90 (5.00)	0.13
Whole Grains	1 medium slice of bread (1 oz) 6 inch tortilla 1 oz tortilla chips 1 small roll 1/2 cup cooked grains or pasta 1 oz dry cereal 1/2 cup hot cereal 1 oz crackers	2.23 (2.48)	2.41 (2.48)	1.45 (2.42)	0.15

Values reported as average serving size equivalents (standard deviation).

## Appendix B

**Table A3:** Maternal–fetal dyad associations with fetal CHD: multivariable analyses.

Characteristic	*n*	Multivariable OR (95% CI)	*p*-Value
Diet Quality			
Diet ID Score	94	1.14 (0.14, 9.27)	0.9
HEI	94	0.94 (0.74, 1.18)	0.59
Food Security	100		
Food Secure		Reference	
Low Food Security		0.36 (0.01, 5.73)	0.49
Very Low Food Security		0.37 (0.01, 5.53)	0.50
Prenatal Vitamin	100		
No		Reference	
Yes		0.21 (0.02, 1.92)	0.17
Diabetes	100		
No		Reference	
Yes		0.72 (0.07, 5.90)	0.77
Body Mass Index	94	0.99 (0.90, 1.08)	0.77
Gestational Age	100	1.36 (1.15, 1.65)	<0.01
Race	100		
Black		Reference	
Multiracial/Other		0.41 (0.02, 6.77)	0.54
White		1.71 (0.24, 15.3)	0.61
Ethnicity	98		
Hispanic/Latina		Reference	
Not Hispanic/Latina		0.23 (0.02, 2.03)	0.19
Marital Status	100		
Div/Sep		—	—
Living with Partner		—	—
Married		—	—
Single		—	—
Education	100		
Four Year		—	—
High School		—	—
Less than HS		—	—
Postgrad		—	—
Technical/Trade		—	—
Two Year		—	—
Employment	99		
Full-time		—	—
Other (Unemployed)		—	—
Part-time		—	—
Seeking Employment		—	—
Stay-at-Home Parent		—	—
Student		—	—
Income ($k)	98		
<15		—	—
15–29		—	—
30–44		—	—
45–59		—	—
60–79		—	—
80–99		—	—
100–149		—	—
>150		—	—
Age (years)	100	1.01 (0.86, 1.18)	0.92
